# Orthostatic Hypotension and Concurrent Autonomic Dysfunction: A Novel Complication of Lung Transplantation

**DOI:** 10.1155/2022/3308939

**Published:** 2022-03-03

**Authors:** Deepika Razia, Sofya Tokman, Sharjeel Israr, Hesham Mohamed, Hesham Abdelrazek, Bhuvin Buddhdev, Ashwini Arjuna, Kendra McAnally, Samad Hashimi, Michael A. Smith, Ross M. Bremner, Rajat Walia, Ashraf Omar

**Affiliations:** ^1^Norton Thoracic Institute, St. Joseph's Hospital and Medical Center, Phoenix, AZ, USA; ^2^Creighton University School of Medicine, Phoenix Regional Campus, Phoenix, AZ, USA

## Abstract

**Background:**

Persistent orthostatic hypotension (OH) is a lesser-known complication of lung transplantation (LTx). In this retrospective case series, we describe the clinical manifestations, complications, and treatment of persistent OH in 13 LTx recipients.

**Methods:**

We identified LTx recipients who underwent transplantation between March 1, 2018, and March 31, 2020, with persistent symptomatic OH and retrospectively queried the records for clinical information.

**Results:**

Thirteen patients were included in the analysis, 9 (69%) had underlying pulmonary fibrosis, and 12 (92%) were male. The median age, height, and body mass index at LTx were 68 years, 70 inches, and 27 kg/m^2^, respectively. Six (46%) patients were deceased at the time of chart abstraction with a median (IQR) posttransplant survival of 12.6 months (6, 21); the 7 remaining living patients were a median of 19.6 months (18, 32) posttransplant. Signs and symptoms of OH developed a median of 60 (7, 75) days after transplant. Patients were treated with pharmacological agents and underwent extensive physical therapy. Most patients required inpatient rehabilitation (*n* = 10, 77%), and patients commonly developed comorbid conditions including weight loss, renal insufficiency with eGFR <50 (*n* = 13, 100%), gastroparesis (*n* = 7, 54%), and tachycardia-bradycardia syndrome (*n* = 2, 15%). Falls were common (*n* = 10, 77%). The incidence of OH in LTx recipients at our center during the study period was 5.6% (13/234).

**Conclusions:**

Persistent OH is a lesser-known complication of LTx that impacts posttransplant rehabilitation and may lead to comorbidities and shortened survival. In addition, most LTx recipients with OH at our center were tall, thin men with underlying pulmonary fibrosis, which may offer an opportunity to instate pretransplant OH screening of at-risk patients.

## 1. Introduction

Lung transplantation (LTx) is a lifesaving procedure for patients with end-stage lung disease; however, it is associated with several complications that impact both quality of life and longevity. Persistent orthostatic hypotension (OH) is an unusual complication of LTx with significant clinical implications. OH is defined as a reduction of systolic blood pressure (SBP) of at least 20 mmHg or diastolic blood pressure (DBP) of at least 10 mmHg within 3 minutes of standing and/or an increase in heart rate ≥20 beats/minute but not exceeding the upper limit of normal [1, 2]. OH may result from an inadequate response to postural changes in blood pressure [1, 2] and may lead to cerebral hypoperfusion with resultant dizziness, visual disturbances, and syncope; muscular hypoperfusion with resultant myalgia; and renal hypoperfusion with resultant oliguria. It is also associated with nonspecific symptoms such as weakness, lethargy, fatigue, and falls [3].

OH may also be part of a clinical syndrome of autonomic dysfunction (AD), which is characterized by cardiac, gastrointestinal, and genitourinary disturbances. AD may present as arrhythmias (tachycardia-bradycardia syndrome and atrial fibrillation), ileus, Ogilvie syndrome, gastroparesis, urinary complaints (retention, hesitancy, dysuria, and incontinence), and bowel complaints (diarrhea and constipation) [3].

Physical therapy and rehabilitation are key components of LTx recovery [4]. Despite near-normal lung function, exercise intolerance and reduction in quality of life (QOL) often persist years after transplantation [5–14]. Muscle dysfunction, inactivity, deconditioning, and nutritional depletion can affect exercise capacity before LTx, and an extended hospital and intensive care unit (ICU) stay may impact lung recipients' recovery in terms of exercise tolerance and QOL [15]. Physical rehabilitation after transplantation can improve exercise capacity and reduce the risk of osteoporosis and muscle dysfunction [16]. OH and other symptoms of AD impact a patient's ability to participate in rehabilitation, hence limiting a successful recovery after LTx.

Persistent OH, alone or in conjunction with AD, can lead to significant comorbidities after LTx and can also impair posttransplant rehabilitation. In a pilot sample of 30 consecutive stable LTx recipients from the University of Zurich, the prevalence of undiagnosed OH was 30% (*n* = 9), 3 of these patients were symptomatic, and another 3 had supine hypertension [17]. Although markers of AD as prognostic indicators have been studied in chronic pulmonary diseases and LTx recipients [18–22], the morbidity associated with OH and AD has not been described. In this retrospective case series, we describe the clinical course of 13 LTx recipients with persistent OH.

## 2. Methods

Institutional review board approval with a waiver of patient consent was obtained for this study (PHXU-21-500-137-73-18, dated March 31, 2021). Patients who underwent LTx between March 1, 2018, and March 31, 2020, at Norton Thoracic Institute, Phoenix, Arizona, with persistent symptomatic OH any time after LTx were included. OH was defined as a reduction of SBP of at least 20 mmHg or a reduction of DBP of at least 10 mmHg within 3 minutes of standing and/or an increase in heart rate ≥20 beats/minute but not exceeding the upper limit of normal [1, 2]. Of note, only LTx recipients with ongoing postural dizziness and falls at home, clinic, or hospital were screened and diagnosed with persistent OH. A diagnosis of persistent OH triggered the evaluation of symptoms of AD [3]. Demographic characteristics, medical history, and clinical outcomes were reviewed for all included patients. Gastroparesis was noted on scintigraphy studies before and after LTx. The six-minute walk distance closest to the time of LTx was reported.

### 2.1. Immunosuppression

Induction therapy included a high-dose corticosteroid (methylprednisolone) before perfusion of each lung allograft and antilymphocyte therapy with basiliximab, rituximab, or antithymocyte globulin (ATG). At our center, the vast majority of patients are induced with basiliximab, with the exception of highly sensitized patients and patients undergoing retransplant who developed allograft failure as a result of recurrent or persistent acute cellular or antibody-mediated rejection. Highly sensitized patients typically receive rituximab or ATG at the time of transplant; whereas, patients undergoing retransplant commonly receive ATG. The maintenance immunosuppressive regime was uniform across the study period and consisted of a corticosteroid (prednisone), an antiproliferative agent (mycophenolate mofetil or mycophenolic acid), and a calcineurin inhibitor ((CNI), tacrolimus or cyclosporine). A selective T cell costimulation blocker (belatacept) was used to reduce CNI-induced nephrotoxicity in patients with progressive and advanced chronic kidney disease.

### 2.2. Statistics

Descriptive statistics were used. Median and interquartile range (IQR) were reported for continuous variables. Frequencies and percentages were reported for categorical variables. The Kaplan–Meier method and log-rank test were used for comparison of survival estimates between included subjects and a control group of remaining subjects who underwent LTx during the study period. Analyses were performed with Stata Statistical Software, Release 13 (Stata Corp College Station, TX).

## 3. Results

During the study period, 234 patients (137 males) with a median (IQR) age of 67.2 years (59.2, 70.5) underwent LTx at our center.

### 3.1. Pre-LTx Clinical Features

Thirteen patients with post-LTx OH were included in this series. The incidence of OH in LTx recipients at our center during the study period was 5.6% (13/234). The median age of the study cohort at the time of LTx was 68 (66, 71) years, and 92.3% (*n* = 12) were male. Median height and body mass index (BMI) at LTx were 70 (68, 72.5) inches and 26.6 (22.8, 28.9) kg/m^2^, respectively; however, 2 of the patients had a BMI <18.5 kg/m^2^. Most patients had idiopathic pulmonary fibrosis (69.2%; *n* = 9), and the remaining patients had a either chronic obstructive pulmonary disease (23.1%; *n* = 3) or combined pulmonary fibrosis and emphysema (7.7%; *n* = 1) (Table 1).

All patients were functionally impaired prior to LTx; New York Heart Association Grade III functional class was observed in 84.6% (*n* = 11) of patients and grade IV was observed in 15.4% (*n* = 2). Median oxygen requirement at rest was 3 (3, 6.5) liters per minute, and 15.4% (*n* = 2) of patients required continuous positive airway pressure for sleep apnea. The median pre-LTx forced expiratory volume in one second (FEV1) was 45% (23, 59), and the median forced vital capacity (FVC) was 49% (40, 61); the lowest FEV1 was 16% of predicted, and the lowest FVC was 18% of predicted. Pre-LTx hypercapnia with nonobstructive pathology was seen in 30.8% (*n* = 4) of patients. None of the patients had valvular heart disease.

Although systemic hypertension was common (61.5%, *n* = 8), only 1 patient carried a pre-LTx diagnosis of OH and was treated with midodrine. Pre-LTx arrhythmias were common (30.8%, *n* = 4) with atrial fibrillation (AFib) in 23.1% (*n* = 3) of patients and left bundle branch block in 7.7% (*n* = 1). Pre-LTx diabetes was present in 30.8% (*n* = 4) of patients; 23.1% (*n* = 3) had peripheral neuropathy, and 1 patient was insulin-dependent. Gastroparesis was present in 15.4% (*n* = 2) of patients, one of whom was diabetic. Osteopenia or osteoporosis was present in 30.8% (*n* = 4) of patients, and 15.4% (*n* = 2) had chronic kidney disease (stage III).

### 3.2. Peri-LTx Clinical Features

Most patients underwent bilateral LTx (92.3%, *n* = 12). Cardiopulmonary bypass was used in 1 patient and extracorporeal membrane oxygenation in 2 patients. Concomitant left atrial appendage clipping was performed in 15.4% (*n* = 2) of patients. A thoracic epidural catheter was placed before induction of anesthesia uneventfully in 76.9% (*n* = 10) of patients. Intraoperative arrhythmias were seen in 38.5% (*n* = 5) of patients: AFib needing cardioversion in 23.1% (*n* = 3), supraventricular tachycardia in 7.7% (*n* = 1), and ventricular tachycardia in 7.7% (*n* = 1). Intraoperative right ventricular function and pulmonary vein velocity were normal in all patients. Median blood loss was 1 (0.6, 2.3) liter, and blood products were transfused in 46.2% (*n* = 6) of patients.

Four patients (30.8%) developed grade 3 primary graft dysfunction (PGD), and the median time to extubation of these patients was 6 days (4, 7); 1 of these patients had a tracheostomy for prolonged mechanical ventilation. The median time to extubation was 4 days (1, 6) in the entire cohort.

Most patients developed cardiac arrhythmia in the immediate postoperative period. Nine patients (69.2%) developed AFib with rapid ventricular response within the first postoperative week, which was managed with antiarrhythmic agents and cardioversion. A dual-chamber pacemaker was placed in 1 patient 2 weeks after LTx, and radiofrequency ablation was performed in 1 patient 5 months after LTx.

Protracted post-LTx hospitalization was common. All patients (*n* = 13) required a >1-week stay in the ICU; the median duration of hospitalization was 40 days (23, 47), and 76.9% (*n* = 10) of patients were discharged to an inpatient acute rehabilitation facility. Readmission within the first year of LTx was common; 84.6% (*n* = 11) had ≥1 readmission within the first year, with a median number of 2 readmissions. The median duration of readmission hospital stay was 18 days (13, 29), and 3 patients required readmission to an inpatient acute rehab facility.

### 3.3. Post-LTx Clinical Features

OH was typically diagnosed early in the post-LTx course with 12 patients (92.3%) diagnosed within 3 months of transplant. The median decrease in SBP and DBP from supine to standing position was 56 (38, 66) and 25 (10.5, 40) mmHg, respectively. Patients commonly reported dizziness (84.6%, *n* = 11), limited ambulation (76.9%, *n* = 10), falls (76.9%, *n* = 10), and muscle weakness (69.3%, *n* = 9). Patients also commonly developed comorbid conditions including weight loss (median decrease in BMI compared to pre-LTx BMI was 2.7 kg/m^2^) and renal insufficiency with eGFR <50% (*n* = 13, 100%). Fractures sustained during mechanical fall episodes occurred in 46.2% (*n* = 6) of patients, and 38.5% (*n* = 5) required orthopedic surgery. Deconditioning and failure to thrive were present in all patients (*n* = 13) with decubitus ulcers in 15.4% (*n* = 2). Clinical depression was treated in 30.8% (*n* = 4) of patients, and 1 patient had generalized anxiety related to fear of syncope.

**Figure 1:**
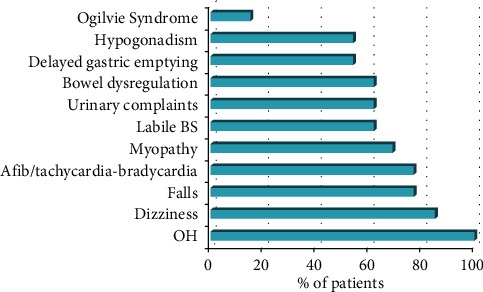
Prevalence and presentation of autonomic dysfunction in lung transplant recipients. OH, orthostatic hypotension; BS, blood sugar.

All patients with OH (*n* = 13) also had additional symptoms of AD that could not be attributed to OH alone. The presentation of AD included urinary complaints (retention, dysuria, incontinence, and hesitancy; 61.5%, *n* = 8), bowel dysregulation (diarrhea (61.5%, *n* = 8), gastroparesis (53.8%, *n* = 7), ileus (100%, *n* = 13), Ogilvie syndrome with cecal dilation >10 cm (15.4%, *n* = 2), labile blood glucose levels requiring frequent insulin dose adjustments (61.5%, *n* = 8), tachycardia-bradycardia syndrome (15.4%, *n* = 2), sweating paroxysms with new-onset peripheral neuropathy (7.7%, *n* = 1), and dry mouth with oral mucosal fragility (7.7%, *n* = 1) (Table 2; Figure 1).

Testosterone levels were measured in 8 male patients, 7 of whom had hypogonadism with a median level of total testosterone, bioavailable testosterone, and free testosterone of 151 ng/dL, 59 ng/dL, and 25 pg/mL, respectively. Six (46%) of the patients were deceased at the time of chart abstraction with a median posttransplant survival of 12.6 months (6, 21); 7 living patients were a median of 19.6 months (18, 32) posttransplant. Follow-up was available for a median of 17.7 (14.2, 29.6) months, and median survival was 17.4 (9.2, 23.2) months.

Pharmacologic and mechanical agents were used to treat OH. Commonly used medications included midodrine (100%, *n* = 13), fludrocortisone (53.8%, *n* = 7), and droxidopa (53.8%, *n* = 7; Table 3). These medications were used in an escalating fashion with midodrine started first, fludrocortisone added second, and droxidopa added third for treatment of refractory patients who could obtain insurance approval for this medication. Drug side effects were common and included supine hypertension (76.9%, *n* = 10), fluid retention, and hyponatremia, with the latter two particularly common among patients treated with fludrocortisone. In an attempt to reduce tacrolimus-induced OH, 1 patient was transitioned to a cyclosporine-based regimen and 1 patient was treated with belatacept, which allowed us to target lower tacrolimus troughs. Compression socks, TED hose, and abdominal binders were used in all patients. A port for outpatient administration of intravenous fluids on a biweekly basis was placed in 1 patient. Despite ≥2 treatment strategies, orthostatic signs were challenging to treat in 53.8% (*n* = 7) of patients. Four patients were on triple therapy (midodrine, fludrocortisone, and droxidopa); none of these 4 patients were alive at the end of the study period. Of those who were alive (*n* = 7), OH was self-limiting in 1 patient and recalcitrant to therapy in 6 patients who still require some form of treatment.

**Figure 2:**
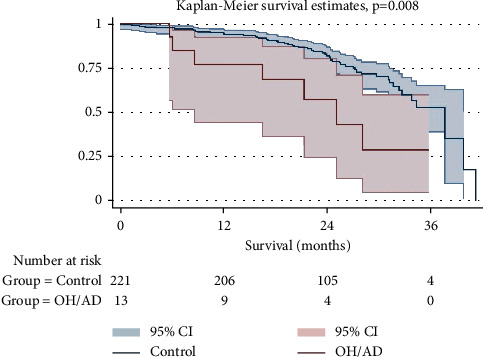
Kaplan–Meier survival analysis stratified by subjects with posttransplant orthostatic hypotension and/or autonomic dysregulation and a control group of remaining subjects transplanted during the study period. OH, orthostatic hypotension; AD, autonomic dysregulation; CI, confidence interval.

Last, the Kaplan–Meier survival analysis between the study group and the remaining LTx recipients during the study period was conducted. The probability of survival at 3 years was significantly lower in the study group than in the control group (46.2% vs. 74.8%, *p*=0.008, Figure 2). The median (IQR) survival of the study group and controls was 25.1 (16.5, 33.7) months and 37.8 (32.5, 43.1) months, respectively.

## 4. Discussion

We identified 13 LTx recipients at our center with OH; 100% (*n* = 13) had additional symptoms of AD with resultant significant morbidity and mortality. The etiology of OH and AD after LTx remains unclear; however, several hypotheses have been proposed. One hypothesis focuses on vagus nerve injury. The right and left vagus nerves contribute to the cardiac, pulmonary, and esophageal plexuses and enter the diaphragm through the esophageal hiatus as the posterior and anterior vagal trunks. In light of its course through the thoracic cavity, it may be subject to mechanical, ischemic, and hypothermic injury at the time of transplant, and the manifestations of the injury may vary depending on which branch was affected.

Although intraoperative vagus nerve injury may explain OH and AD in the early post-LTx course, additional factors need to be considered since 7.7% (*n* = 1) developed OH and AD 17 months after surgery. Notably, the patient who developed OH and AD >6 months after transplant was >65 years old. OH is a well-described complication of aging and senescence [23, 24]. An association between age-related frailty and degenerative dysautonomia has been demonstrated in a community-dwelling population of 65–101 years old [24]. In frail older patients, baroreflex sensitivity, *α*-1 adrenergic vasoconstrictor response to sympathetic stimuli, and parasympathetic activity may be altered. In addition, renal salt and water conservation, comorbidity burden, and impaired thirst response to dehydration may be associated with poor homeostasis to orthostatic stress [25]. Furthermore, sarcopenia, neuromuscular frailty, or critical illness myopathy potentially compromise venous return and result in OH.

Additionally, post-LTx medications may contribute to the development of OH. For example, CNIs, the backbone of posttransplant immunosuppressive therapy in solid organ transplant recipients, have been reported to cause peripheral neuropathy. Bhagavati et al. [26] reported 2 patients who developed chronic sensorimotor polyneuropathy after tacrolimus use following renal transplantation. One patient had an unusual presentation with bilateral facial and extremity weakness and a relapsing course. The other patient presented with focal sensory symptoms in one hand. Electrophysiological studies confirmed widespread, predominantly demyelinating or axonal polyneuropathy. Although highly speculative, it is theoretically possible that CNIs have a similar impact on the autonomic nervous system, thereby leading to OH and AD.

Last and notably, the vast majority of LTx recipients with OH were male, raising the possibility that sex hormones play a role in this condition. Testosterone deficiency and hypogonadism have been described after solid organ transplantation. Fleischer et al. [27] measured serum testosterone, estradiol, sex hormone-binding globulin, and gonadotropin levels for the first two years after transplantation in a cohort of 108 heart transplant recipients. Total and free testosterone levels were lowest during the first month (257 ± 131 and 6.2 ± 3 ng/dl, respectively) and normalized by 2 months. Gonadotropins were low in the majority, suggesting hypothalamic–pituitary–gonadal axis suppression. Low total testosterone persisted in 14% of patients at 1 year and 18% at 2 years. Prednisone was the major predictor of serum testosterone. Although there is no obvious mechanism driving both hypogonadism and OH and AD, it is possible that hypogonadism contributes to frailty in male LTx recipients, making blood pressure fluctuations less tolerable.

Although the etiology of OH and AD post-LTx remains unknown, it clearly has a profound impact on QOL, morbidity, and mortality for the LTx recipients that developed this syndrome. Patients with OH were commonly dizzy, weak, and frail and had difficulty with physical rehabilitation due to these symptoms. They suffered from renal failure and recurrent falls, some of which required orthopedic intervention. OH was commonly accompanied by additional symptoms of AD, including urinary complaints (retention, dysuria, incontinence, and hesitancy), diarrhea, labile blood glucose levels, tachycardia-bradycardia syndrome, gastroparesis, ileus, Ogilvie syndrome with cecal dilation >10 cm, sweating paroxysms with new-onset peripheral neuropathy, and dry mouth with oral mucosal fragility (Table 2; Figure 1). These symptoms likely exacerbated an already tenuous clinical state and almost certainly contributed to fragility and poor QOL. Similar morbidity associated with OH has been described in other solid organ transplant recipients. Nygaard et al. [28] described dizziness and poor functional status in heart transplant recipients; Kuten et al. [29] reported multiple readmissions in pancreas transplant recipients; and Khurana et al. [30] and Munjal et al. [31] reported urinary complaints and hypertensive hemorrhagic infarction, accordingly, in pancreas-kidney transplant recipients.

Persistent OH can be treatment refractory despite the use of both pharmacological and mechanical therapies. Our standard protocol is to rule out adrenal insufficiency and use intravenous fluids, mechanical strategies (such as TED hose and abdominal binders), midodrine, fludrocortisone, and droxidopa on a case-to-case basis, along with outpatient physiotherapy. Escalating doses of medications and polypharmacy may lead to untoward side effects including supine hypertension, edema, and electrolyte disturbances. Mechanical devices such as TED hose may be difficult to put on, particularly by weak patients, and abdominal binders may restrict breathing and food intake. The complications of AD are also difficult to treat. For example, gastroparesis often required multiple interventions, including endoscopy with pyloric dilation and botulinum toxin injection, and patients are often left malnourished, nauseous, and feeding tube dependent.

Our report has several limitations. First, it is a descriptive, single-center case series with low levels of evidence. Nevertheless, to the best of our knowledge, this is the first series in the LTx literature describing OH and AD as possible complications of LTx. Second, we have likely underestimated the incidence of post-LTx OH and AD as patients are not routinely screened for these conditions, and therefore, minimally symptomatic patients were not identified. Third, since the clinical presentation of AD is so variable, it is difficult to differentiate between symptoms attributable to AD versus those attributable to other etiologies. However, despite these challenges, the syndrome of OH and AD is worthy of formal description in the literature as well as ongoing study as it has a profound impact on post-LTx morbidity and mortality. In fact, as a result of these observations after LTx, our center has incorporated a formal assessment of orthostasis as part of the standard pre-LTx evaluation. However, further large studies are needed to better define this syndrome and its impact on the clinical outcomes of lung transplant recipients.

## 5. Conclusions

OH with concurrent AD is a newly described complication of LTx that leads to significant comorbidities, impairs post-LTx rehabilitation, and may be associated with shortened post-LTx survival. The majority of LTx recipients with OH at our center were tall, thin men with underlying pulmonary fibrosis, which may offer an opportunity to instate pretransplant OH screening of at-risk patients. Further studies are needed to identify risk factors for OH and AD as well as to identify the etiology and pathophysiology of this syndrome.

## Figures and Tables

**Table 1:** Baseline characteristics, pretransplant clinical features, and outcomes in the study cohort.

Variables	Study cohort, *n* = 13
Age, years^†^	68 (66, 71)
Gender, male	12, 92.3
Height, inches^†^	70 (68, 72.5)
Body mass index, kg/m^2†^	26.6 (22.8, 28.9)
Lung transplant, bilateral	12, 92.3
Underlying diagnosis	
IPF	9, 69.2
COPD	3, 23.1
CPFE	1, 7.7
Pre-LTx clinical features	
LAS^†^	47.5 (35.4, 54.7)
Creatinine, mg/dL^†^	0.8 (0.8, 1.2)
Distance walked in six minutes, meters^†^	346 (273, 393)
Right atrial pressure, mmHg^†^	7.5 (4, 12.5)
Mean pulmonary artery pressure, mmHg^†^	22 (20, 33)
Pulmonary capillary wedge pressure, mmHg^†^	15 (6, 19)
Cardiac index	3.3 (2.9, 3.7)
Known orthostatic hypotension	1, 7.7
Arrhythmia	4, 30.8
Preexisting peripheral neuropathy	3, 23.1
Preexisting gastroparesis	2, 15.4
Immunosuppression	
Induction: basiliximab	10, 76.9
Induction: rituximab/IVIG	2, 15.4
Induction: ATG	1, 7.7
Mycophenolate mofetil, tacrolimus, steroids	13, 100
Cyclosporin	1, 7.7
Belatacept	1, 7.7
Post-LTx clinical features	
Primary graft dysfunction grade 3	4, 30.8
Time to endotracheal extubation, days^†^	4 (1, 6)
Atrial fibrillation in first postoperative week	9, 69.2
Decrease in SBP supine to standing^†^	56 (38, 66)
Decrease in DBP supine to standing^†^	25 (10.5, 40)
Supine hypertension	10, 76.9
Discharged to home	3, 23.1
Inpatient rehabilitation after LTx	10, 76.9
Readmissions for inpatient rehabilitations	3, 23.1
Number of readmissions in first year^†^	2 (1, 3)
Length of stay for readmissions, days^†^	18 (13, 29)
Deaths	6, 46.2
1^st^ year	3, 23.1
2^nd^ year	2, 15.4
3^rd^ year	1, 7.7

Data expressed as numbers, percentages unless specified otherwise; ^†^data expressed as median (interquartile range). IPF, idiopathic pulmonary fibrosis; COPD, chronic obstructive pulmonary disease; CPFE, combined pulmonary fibrosis and emphysema; LTx, lung transplant; LAS, lung allocation score; IVIG, intravenous immunoglobulin; ATG, rabbit antithymocyte globulin; SBP, systolic blood pressure; DBP, diastolic blood pressure.

**Table 2:** Manifestations of autonomic dysfunction in individual patients by patient ID.

Patient ID	OH	Dizziness	AFib/tachycardia-bradycardia	Myopathy	Ogilvie syndrome	Labile BS	Urinary complaints	Bowel dysregulation	DGE	Falls	Hypogonadism
1	+	+	+	+		+	+	+	+	+	
2	+	+	+	+						+	+
3	+	+	+	+	+		+		+	+	+
4	+	+					+	+			+
5	+	+		+	+	+	+		+	+	+
6	+	+	+	+		+			+		
7	+	+	+			+	+			+	
8	+	+	+			+		+		+	
9	+	+	+	+		+		+		+	
10	+	+	+	+		+	+	+	+		+
11	+		+	+				+	+	+	+
12	+		+				+	+		+	+
13	+	+		+		+	+	+	+	+	

+, present. OH, orthostatic hypotension; AFib, atrial fibrillation; BS, blood sugar; DGE, delayed gastric emptying.

**Table 3:** Treatment strategies for orthostatic hypotension in individual patients by patient ID.

Patient ID	Midodrine	Fludrocortisone	Droxidopa	Desmopressin	Pyridostigmine/neostigmine	Mechanical treatment strategies (compression socks/TED hose/abdominal binders)	Physiotherapy
1	+	+				+	+
2	+	+	+			+	+
3	+	+	+	+	+	+	+
4	+						+
5	+		+			+	+
6	+	+				+	+
7	+		+			+	+
8	+						+
9	+	+	+	+	+	+	+
10	+	+	+			+	+
11	+					+	+
12	+	+				+	+
13	+		+			+	+

+, yes.

## Data Availability

The data used to support the findings of this study are available from the corresponding author upon reasonable request.

## Abbreviations

AD: Autonomic dysfunction
AFib: Atrial fibrillation
ATG: Antithymocyte globulin
BMI: Body mass index
CNI: Calcineurin inhibitor
DBP: Diastolic blood pressure
FEV1: Forced expiratory volume during one second
FVC: Forced vital capacity
ICU: Intensive care unit
IQR: Interquartile range
LTx: Lung transplantation
OH: Orthostatic hypotension
PGD: Primary graft dysfunction
QOL: Quality of life
SBP: Systolic blood pressure.
